# English versus Non-English Language Preference and Computed Tomography Use in Pediatric Minor Head Trauma

**DOI:** 10.5811/westjem.64410

**Published:** 2026-10-26

**Authors:** David R. Nelson, Zachary T. Sheff, Alexander J. Kinstedt, Jennifer R. Barker, Courtney A. Kelly

**Affiliations:** * Peyton Manning Children’s Hospital at Ascension St. Vincent, Pediatric Trauma Department, Indianapolis, Indiana; † Eli Lilly and Company, Indianapolis, Indiana; ‡ Indiana University School of Medicine-Terre Haute, Terre Haute, Indiana; § Peyton Manning Children’s Hospital at Ascension St. Vincent, Department of Emergency Medicine, Indianapolis, Indiana; || Children’s Hospital of Michigan, Division of Emergency Medicine, Detroit, Michigan

## Abstract

**Introduction:**

Whether the use of computed tomography (CT) in pediatric minor head trauma varies by documented language preference (English vs non-English), independent of race, is not well understood. Our objective was to identify factors associated with head CT use among children with minor head trauma in an institutional Pediatric Emergency Care Applied Research Network (PECARN)-based pathway.

**Methods:**

This single-center retrospective cohort study included patients 2 to 14 years of age presenting with minor head trauma, January 2019 to June 2022, who were classified as low, intermediate, or high risk using PECARN criteria. Our primary outcome measure was a three-level clinical pathway: no CT and no traumatic brain injury (TBI); clinical TBI without CT; or CT performed. Secondary outcomes were CT positivity and short-term clinical outcomes. Multinomial logistic regression assessed associations between pathway and language preference, age, headache, weighted observed symptom score (OSS), and race (White vs non-White).

**Results:**

Among 1,167 patients, 319 (27.3%) underwent CT, 132 (11.3%) had clinical TBI diagnosis without CT, and 716 (61.4%) had neither; 430 (36.8%) were classified intermediate risk—the subgroup in which the imaging decision is most discretionary. Non-English language preference was associated with lower adjusted odds of CT in the intermediate-risk subgroup (adjusted odds ratio [aOR], 0.48; 95% CI, 0.24–0.92; *P* = .03) but not in the full cohort (aOR, 0.78; 95% CI, 0.50–1.22; *P* = .27). Within the intermediate-risk subgroup, OSS was strongly associated with CT use (aOR, 3.65; 95% CI, 2.67–5.00; *P* < .001) and headache with clinical TBI without CT (aOR, 4.94; 95% CI, 1.64–14.90; *P* = .005), whereas race was not independently associated with CT use (aOR, 1.36; 95% CI, 0.82–2.25; *P* = .48). Language preference was not associated with CT positivity among scanned patients (aOR, 1.10; 95% CI, 0.44–2.72; *P* = .84), and short-term outcomes did not differ by language (all *P* > .35).

**Conclusion:**

Documented non-English language preference was independently associated with lower use of head CT in PECARN intermediate-risk children; race was not after adjustment for clinical severity. Yield and short-term outcomes did not differ by language preference among imaged patients; however, imaging selection precludes conclusions about mechanism.

## INTRODUCTION

Head computed tomography (CT) is the standard diagnostic tool for pediatric minor head trauma and a leading source of ionizing radiation exposure in children; however, whether its use is distributed equitably across patients of different races and non-English-speaking status (English versus non-English primary language at registration) remains poorly understood.^1^–^5^ These radiation risks, along with concerns about cost and the potential need for sedation in younger patients, have driven substantial scrutiny of CT overuse.^6^–^10^ The Pediatric Emergency Care Applied Research Network (PECARN) prediction rule stratifies patients into high-risk (CT recommended), intermediate-risk (observation versus CT appropriate), and low-risk (CT not recommended) categories, and has been extensively validated across multiple countries.^11^–^16^ Implementations of PECARN-based pathways have reduced CT use by 20% to 45% without increasing missed traumatic brain injury (TBI) or clinically important TBI (ciTBI).^17^–^21^ Despite this, the intermediate-risk category—where the decision to image or observe is left to clinical judgment informed by physician experience, symptom severity, worsening during observation, and parental preference—remains a locus of substantial practice variation.^22^,^23^

Prior studies have identified racial and ethnic differences in cranial CT use for pediatric head trauma, with White children generally more likely to receive imaging, particularly at lower risk levels.^24^–^26^ Similar disparities have been reported in pediatric ED imaging broadly^27^ and in concussion diagnosis and evaluation.^28^,^29^ However, none of these studies examined documented non-English language preference as a factor independently associated with imaging decisions. A growing body of evidence has linked limited English proficiency (LEP) to poorer communication quality, lower caregiver trust, and worse process measures in pediatric EDs, including higher revisit rates and delays in care.^30^–^33^ Prior work has shown that language barrier—rather than race or ethnicity—was the greatest driver of disparities in a pediatric ED, with Spanish-speaking families receiving fewer interventions including imaging.^34^ The intermediate-risk imaging decision is particularly communication-dependent, relying on caregiver-reported symptom evolution and parental preference, raising the possibility that language barriers may specifically affect this decision in ways not captured by prior racial disparity analyses.

Our objective was to identify clinical and demographic factors associated with the use of head CT in pediatric patients 2 to 14 years of age presenting with minor head trauma to a single pediatric emergency department (ED), with a specific focus on whether documented non-English language preference was independently associated with the imaging decision after adjustment for clinical severity. We used a multinomial outcome framework distinguishing three clinical pathways (no CT/no TBI; clinical TBI without CT; CT performed) to assess associations with baseline clinical and demographic variables.

**Population Health Research Capsule**

What do we already know about this issue?

*Prior pediatric head computed tomography (CT) studies use binary CT/no-CT outcomes; language-associated differences are understudied.*

What was the research question?

*Do English vs non-English language preference and race predict CT, clinical traumatic brain injury (TBI) without CT, or neither?*

What was the major finding of the study?

*Intermediate-risk children: non-English preference had lower CT odds (aOR, 0.48; 95% CI, 0.24–0.92; P = .03).*

How does this improve population health?

*This study identifies language-associated variation in discretionary CT use, motivating prospective evaluation of communication supports.*

## METHODS

### Study Design and Setting

This was a retrospective observational study of pediatric patients presenting to a single tertiary care pediatric ED with minor head trauma between January 1, 2019 and June 30, 2022. The institution received Level I pediatric trauma center accreditation in September 2021. The cohort was originally assembled to evaluate CT use surrounding this transition; because the pre- and post-comparison showed no difference in overall CT use, the present analysis examines clinical-pathway variation within the full predefined cohort. Patient-level data were retrospectively abstracted from the electronic health record and managed using Research Electronic Data Capture.^35^,^36^ This study adhered to the methodologic standards for emergency medicine medical record review research described by Worster and colleagues^37^—including abstractor training, case selection criteria, variable definition, abstraction form structure, performance monitoring, blinding to hypothesis, medical record identification, sampling method, missing-data management, and institutional review board approval (Supplemental Methods).

### Study Population

All patients 2 to 14 years of age who presented to the ED with a primary ICD-10 diagnosis code corresponding to minor head trauma were eligible for inclusion (Supplemental Table 1). The upper age limit of 14 years reflects the institution’s operational definition of the pediatric trauma population during the study period; patients aged 15 years and older who presented with trauma were evaluated in the adult ED and therefore were outside the sampling frame for this pediatric ED cohort. The qualifying ICD-10 codes align with the Centers for Medicare and Medicaid Services Merit-based Incentive Payment System Clinical Quality Measure for ED CT use in minor blunt head trauma (Quality ID 416).^38^ Patients were excluded if they had a pre-existing ventriculoperitoneal shunt, a history of brain tumor or coagulopathy, a chief complaint unrelated to minor head trauma, or had undergone head CT at an outside facility.

### Risk Classification

Each patient was classified into one of three risk categories for ciTBI—high risk (CT recommended), intermediate risk (observation or CT appropriate), or low risk (CT not recommended)—using criteria from the PECARN prediction rule for children 2 years of age or older (Supplemental Figure 1).^11^ The intermediate-risk category, in which the imaging decision is left to clinical judgment informed by physician experience, symptom severity, worsening during observation, and parental preference, was the primary analytic focus because it represents the population for whom practice variation and communication-dependent factors are most likely to influence the CT decision.

### Variables

The primary outcome was clinical pathway, a three-level variable: (1) no CT and no TBI diagnosis (reference); (2) TBI diagnosed without CT; or (3) CT performed. Independent variables of interest were race (White, Black, Asian, multiracial, other) and language preference (English versus non-English). Covariates included age, sex, Hispanic ethnicity, PECARN risk category, mechanism of injury, and individual PECARN clinical signs (headache, hematoma, emesis, loss of consciousness, severe mechanism of injury). Detailed variable definitions, including the rationale for the three-level outcome, are provided in Supplemental Methods.

### Statistical Analysis

We calculated descriptive statistics for the full cohort and stratified them by PECARN risk category and CT receipt. Continuous variables are reported as means with standard deviations and medians; categorical variables as frequencies with percentages. Group comparisons used Wilcoxon rank-sum, chi-square, or Fisher exact tests, as appropriate.

To identify factors associated with clinical pathway, multinomial logistic regression was employed with the three-level outcome in the full cohort and among intermediate-risk patients, with the no-CT/no-TBI-diagnosis group as the reference category. To reduce multicollinearity and multiplicity, variable selection used a variance preservation approach^39^ to identify the most informative variables, and patients were randomly assigned to two-thirds model-building and one-third model-validation subsets stratified by CT use and TBI diagnosis (further details in Supplemental Statistical Methods). Variables significant in both the building subset (two-tailed *P* < .05) and the validation subset (one-tailed *P* < .05) were retained in the final model. Four objective clinical signs were combined into a weighted observed symptom score (OSS = 2 × emesis + 2 × loss of consciousness + hematoma + severe mechanism of injury; range 0–6) to summarize observable severity independent of verbal report; OSS derivation and internal validation are detailed in Supplemental Statistical Methods. Race was modeled as a binary variable (White versus non-White) to ensure adequate cell sizes in all contrasts; a sensitivity analysis using a multilevel race specification produced consistent findings but yielded unstable estimates in the smallest race categories due to sparse cells. The Cochran-Mantel-Haenszel test assessed the overall association between language preference and binary CT receipt stratified by symptom severity. Results are reported as adjusted odds ratios (aOR) with 95% CIs.

We performed two hypothesis-generating exploratory analyses to describe patterns that might inform possible explanations for the observed association between language preference and CT use; methods are detailed in Supplemental Statistical Methods. Statistical inference for the primary research question is based solely on the multinomial regression. Two-tailed *P* < .05 was used for hypothesis testing. Analyses were performed using Altair Analytics Workbench (Altair Engineering Inc).

## RESULTS

### Study Population and Patient Outcomes

Of 1,348 patients identified, 1,167 met inclusion criteria (Supplemental Figure 2). The 181 excluded were primarily for a chief complaint unrelated to minor head trauma (*n* = 121), outside-hospital CT (*n* = 36), brain tumor or coagulopathy history (*n* = 19), or ventriculoperitoneal shunt (*n* = 5).

Across the full cohort, 643 patients (55.1%) were classified as low risk, 430 (36.8%) as intermediate risk, and 94 (8.1%) as high risk (Figure 1). Use of CT did not differ significantly between the pre-accreditation period (January 2019 to September 2021; 27.2%) and the post-accreditation period (October 2021 to June 2022; 27.9%; *P* = .81), and the full cohort was analyzed as a single group. Among the three pathway groups, 716 patients (61.4%) had neither CT nor clinical TBI diagnosis, 132 (11.3%) had a clinical TBI diagnosis without CT, and 319 (27.3%) underwent CT, of whom 167 (52.4%) had TBI identified. Overall, 299 (25.6%) received a TBI diagnosis, 132 (44.1%) without CT.

### Factors Associated with Computed Tomography Utilization: Full Cohort

Table 1 summarizes demographic and clinical characteristics of the full cohort stratified by CT receipt. Patients who underwent CT were older (mean age 7.45 vs 6.63 years; *P* = .003), more often White (64.3% vs 54.5%; *P* = .003), and less often Black (27.3% vs 35.7%; *P* = .006). Language preference, Hispanic ethnicity, and sex did not differ by CT status (all *P* ≥ .08; Table 1).

Markers of clinical acuity were more common among CT recipients, including high PECARN risk, emesis, loss of consciousness, severe mechanism of injury, headache, and hematoma (all *P* ≤ .002; Table 1), and CT recipients had a higher mean OSS (1.26 vs 0.22; *P* < .001). Hematoma was associated with CT receipt despite not appearing in PECARN intermediate-risk criteria for children 2 years of age or older, consistent with prior reports.^40^–^42^

Clinical TBI diagnosis at the index visit was more common among CT recipients (52.4% vs 15.6%; *P* < .001), as were inpatient admission (5.6% vs 0.6%; *P* < .001) and longer outpatient length of stay (*P* < .001; Table 1). Unplanned readmission did not differ between groups (*P* > .99).

### Patient Characteristics Stratified by Computed Tomography Receipt: Intermediate-Risk Subgroup

Table 2 summarizes characteristics of the 430 patients in the intermediate-risk subgroup. Overall, 180 patients (41.9%) underwent head CT. As in the full cohort, markers of clinical acuity—emesis, loss of consciousness, hematoma, and severe mechanism of injury—were each significantly more common among CT recipients (Table 2). Patients who underwent CT also had a higher mean OSS than nonrecipients (1.68 vs 0.57; *P* < .001). Two reversals from the full-cohort pattern emerged: CT recipients were younger than nonrecipients (7.56 vs 8.50 years; *P* = .01), and headache was more common in the no-CT group (82.0% vs 67.2%; *P* < .001).

Demographic differences were more pronounced than in the full cohort. Patients who underwent CT more often had English language preference (86.7% vs 76.0%; *P* = .006), more often were White (65.0% vs 51.2%; *P* = .004), and less often Black (25.6% vs 41.6%; *P* < .001); Hispanic ethnicity and sex did not differ (*P* ≥ .11; Table 2). Clinical TBI diagnosis, inpatient admission, and outpatient length of stay were each higher among CT recipients; unplanned readmission did not differ (Table 2).

### Multinomial Regression Results

The primary multinomial regression model included OSS, headache, language preference, and age (Figure 2). In both the full cohort and the intermediate-risk subgroup, OSS was strongly associated with CT use (full cohort aOR, 3.67; 95% CI, 3.00–4.50; intermediate-risk aOR, 3.65; 95% CI, 2.67–5.00; both *P* < .001). The OSS was also associated with the clinical TBI diagnosis pathway in the full cohort (aOR, 1.39; 95% CI, 1.04–1.87; *P* = .03) but not in the intermediate-risk subgroup (aOR, 1.15; 95% CI, 0.78–1.70; *P* = .47).

Headache showed a distinct pattern. In both cohorts, headache was strongly associated with the clinical TBI diagnosis pathway (full cohort aOR, 6.95; 95% CI, 4.45–10.86; *P* < .001; intermediate-risk aOR, 4.94; 95% CI, 1.64–14.90; *P* = .005) and with CT use, although with smaller effect sizes (full cohort aOR, 2.88; 95% CI, 1.98–4.19; *P* < .001; intermediate-risk aOR, 2.69; 95% CI, 1.39–5.22; *P* = .003). Among scanned patients, headache was associated with CT positivity in the full cohort (aOR, 2.88; 95% CI, 1.75–4.74; *P* < .001).

Language preference (English vs non-English) was independently associated with CT use in the intermediate-risk subgroup. After adjustment for OSS, headache, and age, patients with documented non-English language preference had lower odds of CT than those with English preference (aOR, 0.48; 95% CI, 0.24–0.92; *P* = .03), an association not observed in the full cohort (aOR, 0.78; 95% CI, 0.50–1.22; *P* = .27). Language preference was not associated with CT positivity in either the full cohort (aOR, 1.03; 95% CI, 0.63–1.70; *P* = .93) or the intermediate-risk subgroup (aOR, 1.10; 95% CI, 0.44–2.72; *P* = .84). Race (White vs non-White)—included as a candidate variable in screening but not retained—was added post hoc to the intermediate-risk regression and was not independently associated with clinical pathway (aOR, 1.36; 95% CI, 0.82–2.25; *P* = .48); the language association was unchanged.

### Clinical Pathway Differences by Language and Symptom Severity

The following two sections report exploratory hypothesis-generating analyses; statistical inference for the primary research question is based solely on the multinomial regression above.

Figure 3 plots observed versus expected clinical pathway proportions for intermediate-risk patients with documented non-English language preference (expected values derived from a model fit to English-preference patients) across OSS levels. At OSS = 0, these patients had a higher observed probability of clinical TBI diagnosis (42.9% vs 27.1% expected) and lower probability of CT (11.9% vs 19.4% expected), although the distribution did not differ significantly (*P* = .20; *n* = 198). At OSS = 2, distributions differed significantly (*P* = .02; *n* = 139); CT was performed in 44.0% vs 71.9% of English-preference patients (*P* = .007). Across strata, the Cochran-Mantel-Haenszel test confirmed an overall association between language preference and CT receipt (common OR, 2.10; 95% CI, 1.17–3.78; *P* = .01; Breslow-Day *P* = .46). These findings should be interpreted cautiously given small subgroup sizes (*n* = 42 at OSS = 0; *n* = 25 at OSS = 2; *n* = 4 at OSS ≥ 3).

### Exploratory Classification Tree Analysis

An exploratory classification tree of intermediate-risk patients is provided in Supplemental Figure 3. The OSS was the most influential feature, and the tree showed lower CT use among patients with documented non-English language preference in selected judgment-dependent branches; however, small terminal-node sample sizes preclude confirmatory inference.

### Clinical Outcomes by Language

Clinical outcomes did not differ significantly by language preference. In the intermediate-risk subgroup, inpatient admission occurred in 0 of 84 patients with documented non-English language preference and 8 of 346 with English preference (2.3%; *P* = .37); unplanned readmission in 1.2% vs 1.7% (*P* > .99); and readmission with a new TBI diagnosis in 1.2% vs 0.6% (*P* = .48). Findings were similar in the full cohort: no significant language-based differences in inpatient admission (1.9% vs 2.0%; *P* > .99), unplanned readmission (0.5% vs 1.2%; *P* = .71), or newly identified TBI on readmission (0.5% vs 0.2%; *P* = .46); overall, newly identified TBI on readmission was rare (3 of 1,167; 0.3%). No measurable differences in short-term clinical outcomes by language preference were identified.

## DISCUSSION

In this study of the use of head CT in pediatric minor head trauma using a multinomial outcome framework distinguishing three clinical pathways, the OSS was strongly associated with CT use, headache was strongly associated with clinical TBI diagnosis without imaging, and documented non-English language preference was independently associated with CT use within the intermediate-risk subgroup—where clinical judgment likely plays the greatest role. Intermediate-risk patients with documented non-English language preference had approximately half the odds of receiving CT after adjustment for clinical severity, whereas race was not independently associated with clinical pathway after the same adjustment. Language preference was not associated with CT positivity (yield) among imaged patients. Because this was a single-center retrospective study and positivity was assessed only among patients selected for imaging, this finding cannot establish the mechanism underlying the observed association with CT use or exclude missed injury among children who did not undergo CT.

### Comparison to Previous Studies

Prior work has documented racial and ethnic differences in cranial CT use for pediatric head trauma, with White children generally more likely to receive imaging at lower risk levels.^24^–^26^ Our unadjusted findings were consistent with these reports. However, after adjustment for measured clinical severity, race was not independently associated with clinical pathway, whereas documented non-English language preference was associated with lower adjusted odds of CT use in the intermediate-risk subgroup. Because race, language preference, clinical presentation, communication, and clinician decision-making cannot be fully disentangled in this retrospective study, this pattern should be interpreted cautiously.

Earlier PECARN work found lower CT use among Black non-Hispanic and Hispanic children in intermediate- and lowest-risk groups, whereas a more recent multicenter prospective analysis found no adjusted differences in head CT use by Hispanic ethnicity, Asian race, or Black race among children aged 2 years and older.^24^,^26^ In the more recent study, lower head CT use among Hispanic children younger than 2 years may reflect disparities in imaging practices.^26^ Our results extend the examination of imaging variation to documented language preference among intermediate-risk children aged 2 to 14 years—the subgroup in which the PECARN rule permits either observation or CT based on clinical judgment.

Among imaged patients, CT positivity did not differ by language preference. However, because CT positivity was assessed only among children selected for imaging, this single-center retrospective analysis cannot determine whether the lower CT-use association reflected appropriate avoidance of imaging, under-imaging, or unmeasured differences in clinical presentation, communication, or clinician decision-making. Prior pediatric ED research has reported differences in caregiver trust and rates of measured interventions by language group, suggesting that language-related factors may contribute to variation in care processes.^34^

### Communication as a Hypothesized Mechanism

The PECARN intermediate-risk decision explicitly depends on four factors: physician experience, symptom number and nature, worsening during observation, and parental preference.^11^ Three of these four rely substantially on verbal communication. A systematic review of pediatric concussion diagnosis found that the most diagnostically specific symptoms—mental fog, noise sensitivity, and nausea—require verbal elicitation rather than direct observation, suggesting that communication barriers may selectively reduce the clinical information available to distinguish between observation and imaging.^43^ When communication is limited, clinicians may rely more heavily on directly observed findings and less on symptom evolution or caregiver-reported concerns—consistent with patients with documented non-English language preference at low symptom burden showing higher clinical TBI diagnosis rates and lower CT rates than expected under English-preference decision patterns (Figure 3). The finding that the language association emerged specifically in the intermediate-risk subgroup—where verbal input may shape the imaging decision more strongly—and was absent in the full cohort is consistent with this hypothesized mechanism. Prior pediatric ED research documents that interpretation during LEP encounters is often intermittent.^44^–^46^ These considerations provide a possible explanation for the observed association; however, the present retrospective data did not measure communication quality, interpreter use, caregiver preference, symptom evolution, or clinicians’ contemporaneous rationale for observation versus CT. Therefore, this mechanism cannot be evaluated directly in this study.

### Strengths

The multinomial outcome framework revealed that a binary CT/no-CT approach conflates two clinically distinct non-CT pathways with different demographic profiles—a distinction not examined in prior PECARN literature.^12^–^21^ The OSS provided a graded measure of objective clinical severity that was more closely associated with CT use than an unweighted symptom count, extending the observation of Bressan and colleagues that PECARN intermediate-risk criteria themselves were not associated with CT decisions.^22^ The multinomial framework distinguished the decision to image from CT yield among those imaged; yield did not differ by language preference. Because imaging was selectively obtained, this analysis cannot establish the mechanism underlying the observed association between language preference and CT use.

### Implications for Practice and Research

If prospective studies confirm that communication-related factors contribute to language-associated variation in CT use, interventions that improve interpreter access beyond initial assessment could be evaluated as a strategy to promote equitable and informed observation versus CT decision-making. Exploratory findings suggest this gap may be most consequential among patients with higher objective severity (OSS ≥ 2), where patients with documented non-English language preference had the highest CT-positive yield despite lower CT use—a pattern that warrants particular attention in interpreter access interventions. Structured reassessment protocols prompting interpreter use at defined intervals during observation could help standardize the information available to clinicians regardless of patient language. Hematoma was independently associated with CT receipt despite not appearing in PECARN intermediate-risk criteria for children 2 years of age and older, consistent with prior reports,^40^–^42^ and may warrant consideration in future guideline refinement.

Future studies should prospectively document interpreter use at each stage of the ED visit—initial assessment, observation, reassessment, and discharge—to directly test the communication mechanism hypothesized here, ideally in multicenter cohorts with larger non-English-language-preference populations and greater linguistic diversity. Additional unmeasured variables include specific language spoken, caregiver health literacy, and physician-level factors such as experience, training level, and language concordance. Clinician workforce composition—including the proportion of attending physicians versus residents, physician assistants, and nurse practitioners—may independently be associated with CT use and the rate of clinical TBI diagnosis without imaging and should be characterized in future multicenter work.

## LIMITATIONS

This study has several limitations. Most importantly, this was a single-center retrospective study; the association observed may not be generalizable to other institutions, patient populations, interpreter service models, or practice environments and should not be interpreted as evidence of a generalized disparity in imaging practice. The finding is best understood as hypothesis-generating and requires confirmation in multicenter prospective studies before inferences about practice elsewhere are warranted. The non-English language-preference group was modest (*n* = 84 in the intermediate-risk subgroup), limiting statistical power for stratified analyses and the precision of within-leaf comparisons in the exploratory classification tree analysis. The documented language-preference variable classified patients as English or non-English but did not capture the specific language spoken, degree of English proficiency, interpreter use during the encounter, or communication nuances during observation.

The three most communication-dependent factors in the PECARN intermediate-risk framework—worsening symptoms during observation, parental preference, and physician experience—were not available in the abstracted dataset and could not be examined directly. The abstracted dataset also did not systematically capture clinicians’ contemporaneous rationale for choosing observation versus CT; therefore, this study cannot determine how individual decision-making processes contributed to the observed imaging patterns. Whether hematoma—independently associated with CT receipt despite not appearing in PECARN intermediate-risk criteria—influences the CT decision through its effect on parental concern or clinician assessment of worsening remains unmeasured and warrants prospective evaluation.

Clinical signs were abstracted as documented in the electronic health record and may not fully reflect information available to the treating physician at the time of the CT decision. Positivity was assessed only among patients selected for imaging and therefore cannot determine whether the lower CT-use association reflected appropriate avoidance of imaging, under-imaging, or missed injury among children who were not imaged. Inter-rater reliability of chart abstraction was not formally tested, although abstractors were trained on operational definitions and abstractor performance was monitored periodically by the research scientist and principal investigator. Although no differences in adverse outcomes by language preference were identified, the study was not powered to detect rare events such as missed ciTBI, and outcomes may have been underestimated if patients sought follow-up outside the health system.

## CONCLUSION

In this single-institution cohort of pediatric patients with minor head trauma, documented non-English language preference was independently associated with the use of head CT among intermediate-risk patients after adjustment for clinical severity, while race was not. Patients with documented non-English language preference had approximately half the adjusted odds of receiving CT in the intermediate-risk subgroup, where PECARN permits observation or CT based on clinical judgment. Positivity and short-term clinical outcomes did not differ by language preference. Because this was a single-center retrospective analysis and clinical signs were abstracted from electronic health record documentation, the findings are associational and hypothesis-generating. The documented signs may not fully capture all information available to the treating clinician at the time of the imaging decision. Accordingly, the findings do not establish that language preference caused differences in imaging use and should not be interpreted as evidence that a similar association is present across other institutions or practice settings. The study also cannot determine the mechanism underlying the observed CT-use association. If confirmed in prospective, multicenter data, interventions to improve interpreter availability throughout the ED observation period would warrant evaluation as a strategy to address this potential disparity.

## Supplementary Information













## Figures and Tables

**Figure 1:**
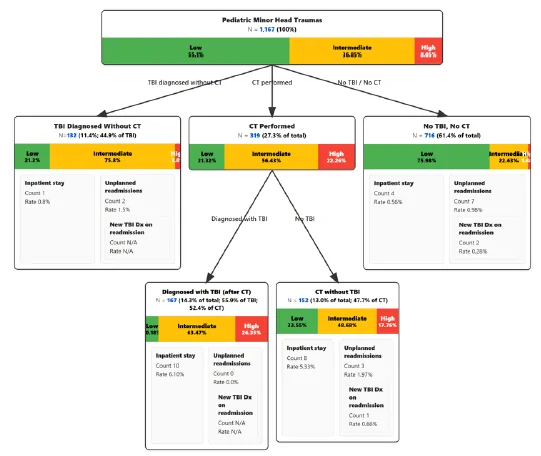
Clinical pathway distribution among 1,167 pediatric patients with minor head trauma at a single pediatric emergency department, 2019–2022. Patients are stratified by Pediatric Emergency Care Applied Research Network risk category (green, low; yellow, intermediate; red, high) and across three clinical pathways. *CT*, computed tomography; *N/A*, zero events observed; *TBI*, traumatic brain injury.

**Figure 2:**
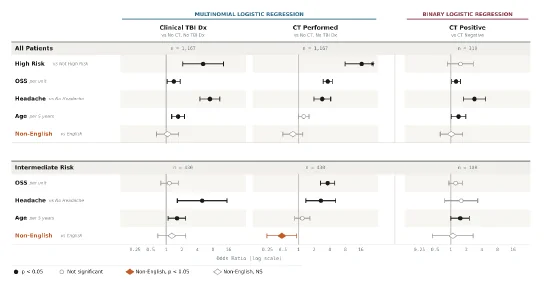
Adjusted odds ratios for multivariable factors associated with clinical pathway among pediatric patients with minor head trauma at a single pediatric emergency department, 2019–2022. Left and center panels show multinomial logistic regression with a 3-level outcome (reference: no CT and no TBI diagnosis); right panel shows binary logistic regression of CT positivity among scanned patients. Upper row: full cohort (*N* = 1,167); lower row: intermediate-risk subgroup (N = 430). Symbol fill and shape indicate significance level as shown in the figure key; diamond markers denote the language preference variable. *CT*, computed tomography; *OSS*, observed symptom score (2 × emesis + 2 × loss of consciousness + hematoma + severe mechanism of injury); *TBI*, traumatic brain injury.

**Figure 3:**
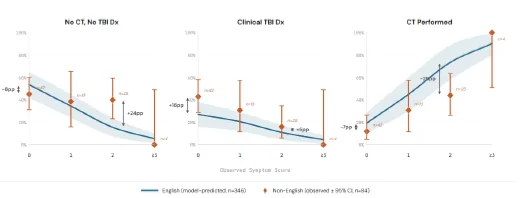
Model-based pathway probability comparisons by observed symptom score (OSS) among intermediate-risk patients with minor head trauma at a single pediatric emergency department, 2019–2022. Blue curves show expected pathway probabilities from a multinomial logistic regression model fit to English-speaking intermediate-risk patients (*n* = 346), evaluated at the covariate profile of non-English-speaking patients. Orange diamonds show observed pathway proportions for non-English-speaking patients (*n* = 84) with 95% CIs. Percentage-point annotations indicate the difference between observed non-English proportions and English-speaker expected values at each OSS level. *CT*, computed tomography; *OSS*, observed symptom score (2 × emesis + 2 × loss of consciousness + hematoma + severe mechanism of injury); *TBI*, traumatic brain injury.

**Table 1:** Demographics, clinical features, and outcomes by head computed tomography receipt in pediatric patients 2 to 14 years of age with minor head trauma.

Variable	Overall (*N* = 1,167)	CT (*n* = 319)	No CT (*n* = 848)	*P* value
Demographics				
Age, years	6.86 (3.83) [6.00]	7.45 (4.02) [7.00]	6.63 (3.73) [6.00]	.003
Sex: female	460 (39.4)	139 (43.6)	321 (37.9)	.08
Race				
White	667 (57.2)	205 (64.3)	462 (54.5)	.003
Black	390 (33.4)	87 (27.3)	303 (35.7)	.006
Asian	15 (1.3)	5 (1.6)	10 (1.2)	.60
Multiracial	54 (4.6)	11 (3.4)	43 (5.1)	.24
Other/unknown	41 (3.5)	11 (3.4)	30 (3.5)	.94
Hispanic ethnicity	149 (13.3)	40 (13.0)	109 (13.4)	.84
English language preference	948 (81.2)	257 (80.6)	691 (81.5)	.72
PECARN risk category				
Low risk	643 (55.1)	68 (21.3)	575 (67.8)	< .001
Intermediate risk	430 (36.8)	180 (56.4)	250 (29.5)	< .001
High risk	94 (8.1)	71 (22.3)	23 (2.7)	< .001
Clinical signs				
Headache	376 (32.2)	158 (49.5)	218 (25.7)	< .001
Hematoma	83 (7.1)	35 (11.0)	48 (5.7)	.002
Emesis	122 (10.5)	92 (28.8)	30 (3.5)	< .001
Loss of consciousness	87 (7.5)	66 (20.7)	21 (2.5)	< .001
Severe mechanism of injury	84 (7.2)	51 (16.0)	33 (3.9)	< .001
OSS	0.50 (0.92) [0.00]	1.26 (1.21) [1.00]	0.22 (0.56) [0.00]	< .001
Mechanism of injury				
Fall	682 (58.4)	203 (63.6)	479 (56.5)	.03
MVC	80 (6.9)	24 (7.5)	56 (6.6)	.58
Assault	26 (2.2)	13 (4.1)	13 (1.5)	.009
Pedestrian struck	4 (0.3)	2 (0.6)	2 (0.2)	.30
Bicycle fall	23 (2.0)	7 (2.2)	16 (1.9)	.74
Other	352 (30.2)	70 (21.9)	282 (33.3)	< .001
Outcomes				
Admitted as inpatient	23 (2.0)	18 (5.6)	5 (0.6)	< .001
TBI at initial visit	299 (25.6)	167 (52.4)	132 (15.6)	< .001
Unplanned readmission	12 (1.0)	3 (0.9)	9 (1.1)	> .99
Outpatient LOS, hours (*n* = 1,144)	3.45 (5.54) [2.55]	5.16 (6.97) [3.65]	2.84 (4.78) [2.32]	< .001
Inpatient LOS, days (*n* = 23)	3.84 (6.91) [1.00]	4.62 (7.66) [1.00]	1.03 (0.82) [0.75]	.20

Continuous variables reported as mean (SD) [median]; categorical variables as n (%).

*CT*, computed tomography; *LOS*, length of stay; *MVC*, motor vehicle collision; *OSS*, observed symptom score (2 × emesis + 2 × loss of consciousness + hematoma + severe mechanism of injury); *PECARN*, Pediatric Emergency Care Applied Research Network; *TBI*, traumatic brain injury.

**Table 2:** Demographics, clinical features, and outcomes by head computed tomography receipt in PECARN intermediate-risk pediatric patients.

Variable	Overall (*N* = 430)	CT (*n* = 180)	No CT (*n* = 250)	*P* value
Demographics				
Age, years	8.10 (3.87) [8.00]	7.56 (4.08) [7.00]	8.50 (3.66) [8.00]	.01
Sex: female	193 (44.9)	89 (49.4)	104 (41.6)	.11
Race				
White	245 (57.0)	117 (65.0)	128 (51.2)	.004
Black	150 (34.9)	46 (25.6)	104 (41.6)	< .001
Asian	6 (1.4)	4 (2.2)	2 (0.8)	.24
Multiracial	11 (2.6)	5 (2.8)	6 (2.4)	.81
Other/unknown	18 (4.2)	8 (4.4)	10 (4.0)	.82
Hispanic ethnicity	41 (9.9)	18 (10.3)	23 (9.6)	.82
English language preference	346 (80.5)	156 (86.7)	190 (76.0)	.006
Clinical signs				
Headache	326 (75.8)	121 (67.2)	205 (82.0)	< .001
Hematoma	37 (8.6)	23 (12.8)	14 (5.6)	.009
Emesis	106 (24.7)	77 (42.8)	29 (11.6)	< .001
Loss of consciousness	64 (14.9)	45 (25.0)	19 (7.6)	< .001
Severe mechanism of injury	68 (15.8)	36 (20.0)	32 (12.8)	.04
OSS	1.03 (1.11) [1.00]	1.68 (1.10) [2.00]	0.57 (0.84) [0.00]	< .001
Mechanism of injury				
Fall	233 (54.2)	117 (65.0)	116 (46.4)	< .001
MVC	40 (9.3)	10 (5.6)	30 (12.0)	.02
Assault	13 (3.0)	8 (4.4)	5 (2.0)	.14
Pedestrian struck	4 (0.9)	2 (1.1)	2 (0.8)	> .99
Bicycle fall	7 (1.6)	3 (1.7)	4 (1.6)	> .99
Other	133 (30.9)	40 (22.2)	93 (37.2)	< .001
Outcomes				
Admitted as inpatient	8 (1.9)	7 (3.9)	1 (0.4)	.01
TBI at initial visit	194 (45.1)	106 (58.9)	88 (35.2)	< .001
Unplanned readmission	7 (1.6)	2 (1.1)	5 (2.0)	.70
Outpatient LOS, hours (*n* = 422)	3.57 (4.25) [2.62]	4.79 (5.65) [3.63]	2.72 (2.62) [2.23]	< .001
Inpatient LOS, days (*n* = 8)	6.33 (10.39) [1.53]	6.94 (11.07) [1.00]	2.07 (NA) [2.07]	> .99

Continuous variables reported as mean (SD) [median]; categorical variables as n (%).

*CT*, computed tomography; *LOS*, length of stay; *MVC*, motor vehicle collision; *OSS*, observed symptom score (2 × emesis + 2 × loss of consciousness + hematoma + severe mechanism of injury); *TBI*, traumatic brain injury.
