# Supplementary material for: English versus Non-English Language Preference and Computed Tomography Use in Pediatric Minor Head Trauma
Source: West J Emerg Med. 2026 Oct 26;27(6):1660–70. doi: 10.5811/westjem.64410 (PMC13762077; doi:10.5811/westjem.64410)
Supplement: Supplementary file 1 [file wjem-27-1660-s001.docx]

**SUPPLEMENTAL METHODS**

**Chart Review Methodology — Detailed Adherence to Worster Criteria**

This study followed the twelve criteria for medical record review research articulated by Worster and colleagues.³⁷

(1) **Abstractor training.** Chart abstractors were second- and third-year medical students from a partner medical school who were not study authors. Abstractors received training on the abstraction protocol and operational definitions before initiating chart review.

(2) **Case selection criteria.** Inclusion and exclusion criteria were defined a priori in the IRB-approved protocol (see main manuscript Methods, Study Population subsection).

(3) **Variable definition.** Variables were defined in advance, with operational definitions for clinical signs aligned to the PECARN criteria.¹¹

(4) **Abstraction form.** Abstractors used a structured REDCap data abstraction form with controlled vocabularies and validated entry checks.³⁵,³⁶

(5) **Performance monitoring.** Abstractor performance was monitored through periodic review of completed records by the research scientist and principal investigator.

(6) **Blinding to hypothesis.** Abstractors were blinded to the hypotheses of the present analysis. The IRB-approved protocol scoped only the pre/post evaluation of CT utilization, sensitivity and negative predictive value of the institutional pathway, and patient demographic characterization, with no analysis of language preference or race specified. The analyses of language preference and race reported here represent a secondary analysis of the prospectively assembled dataset, undertaken by investigators distinct from the chart abstractors after data collection was complete.

(7,8) **Inter-rater reliability.** Inter-rater reliability was not formally tested. This is acknowledged in the Limitations section of the main manuscript.

(9) **Medical record identification.** The medical record source was a single institution's electronic health record, with all eligible records identified through structured ICD-10 queries.

(10) **Sampling method.** The sampling method was complete enumeration of all eligible patients during the 42-month study period rather than random or convenience sampling.

(11) **Missing data.** Patients missing data for any analytic variable were excluded per protocol; the proportion excluded for this reason was small (5 of 188 total exclusions).

(12) **IRB approval.** The study was approved by the institution's Institutional Review Board.

**Variables — Detailed Definitions**

The primary outcome was the clinical pathway observed at the index visit, defined as a three-level variable: (1) no CT performed and no TBI diagnosis (reference category); (2) TBI diagnosed without CT; or (3) CT performed (regardless of result). TBI diagnosed without CT was defined as an encounter in which the treating clinician documented a diagnosis of traumatic brain injury or concussion during the index visit without a head CT being performed, as abstracted from the electronic health record. The three-level outcome was chosen because the binary CT/no-CT framework conflates two clinically distinct non-CT populations — patients discharged without concern for TBI and those diagnosed through clinical assessment alone — that carry different implications for understanding how demographic factors shape the imaging decision.

Independent variables of interest were patient race (White, Black, Asian, Multiracial, Other) and preferred language (English versus non-English), defined as whether the patient's or caregiver's primary language was English, as recorded at registration. Covariates included age (in years), sex, Hispanic ethnicity, PECARN risk category (low, intermediate, high), mechanism of injury (fall, motor vehicle collision, assault, pedestrian struck, bicycle fall, other), and individual PECARN clinical signs — headache, hematoma, emesis, loss of consciousness, and severe mechanism of injury — each abstracted as documented by the treating clinician at the index visit.

**Statistical Methods**

**Variable Screening: Variance Preservation**

Initial variable screening was performed using variance preservation optimization, a dimension-reduction approach that identifies variables accounting for the greatest proportion of outcome variability while minimizing redundancy among selected variables.^39^ The candidate variable set included age, sex, preferred language, race, Hispanic ethnicity, PECARN risk category, mechanism of injury, and the five individual clinical signs (headache, hematoma, emesis, loss of consciousness, severe mechanism of injury). The three-level clinical pathway outcome was specified as the supervised target. This screening step reduced the candidate variable set prior to regression modeling; statistical inference is based on the subsequent multinomial logistic regression, not on the screening procedure.

**Observed Symptom Score: Derivation**

In preliminary multinomial logistic regression models fit to the two-thirds model-building sample, four clinical signs — emesis, loss of consciousness, hematoma, and severe mechanism of injury — each was associated with CT utilization in the same direction and with similar patterns of significance, while headache did not follow this pattern. To reduce dimensionality, these four signs were combined into the Observed Symptom Score (OSS = 2×emesis + 2×loss of consciousness + hematoma + severe mechanism of injury; range 0–6). The 2:1 weighting reflects the approximately twofold larger regression coefficients for emesis and loss of consciousness relative to hematoma and severe mechanism in the building-sample models (Supplemental Table 2). The composite was evaluated in the one-third validation sample, where it showed consistent coefficient direction and equivalent model performance to the four individual components. A secondary model substituting the individual components for the OSS confirmed robustness of results (Supplemental Table 2).

**Exploratory Analysis: Model-Based Pathway Comparisons by Language (Figure 3)**

To explore the mechanism underlying the language-associated disparity, a multinomial logistic regression model was fit to English-speaking intermediate-risk patients only (n = 346) using OSS, headache, and age as variables with the generalized logit link function. Estimated pathway probabilities with 95% confidence intervals were generated using the delta method, evaluated at the covariate profile of the non-English-speaking subgroup (mean age 9.6 years; headache prevalence 85.7%). This model-based approach — estimating expected pathway probabilities for non-English-speaking patients under English-speaker decision patterns — is intended as an exploratory visualization of where observed non-English outcomes diverge from model estimations, not as causal inference. Because the non-English covariate profile falls within the observed range of the English-speaking sample, the estimations do not require extrapolation beyond the support of the training data.

Observed pathway proportions for non-English-speaking patients (n = 84) were calculated at each OSS level (0, 1, 2, ≥3) with 95% Wilson confidence intervals. Chi-square tests compared pathway distributions at each OSS level. The Cochran-Mantel-Haenszel test assessed the overall association between preferred language and binary CT receipt stratified by OSS level; the Breslow-Day test assessed homogeneity of odds ratios across strata.

**Exploratory Analysis: Classification Tree (Supplemental Figure 3)**

A classification and regression tree (CART) was constructed among intermediate-risk patients (n = 430) using entropy as the splitting criterion with cost-complexity pruning. The three-level pathway outcome was modeled as a function of OSS, headache, age (continuous), and non-English speaker status. The final pruned tree (10 terminal nodes, depth 4) was selected by clinical interpretability and minimum cross-validated error. Feature importance was quantified as the relative contribution of each variable to entropy reduction across all splits.

Pathway distributions were compared between the Clinical Judgment Zone (OSS ≤ 1; n = 260) and the Imaging Zone (OSS ≥ 2; n = 170) using chi-square tests. CT-positive yield at each terminal node was compared to the high-risk reference rate (62%) using Dunnett's test. Age threshold and language yield comparisons within specific leaves used Fisher's exact test.

Both exploratory analyses are hypothesis-generating. Statistical inference for the primary research question is based solely on the multinomial regression reported in the main text.

**Model Selection Validation: Cross-Validation Comparison**

To assess whether the multinomial logistic regression model adequately captured the relationship between variables and clinical pathway, or whether nonlinear interactions or complex decision boundaries might improve predictive performance, a 5×10 repeated stratified cross-validation (50 total resamples) was conducted comparing six model types on identical resampling folds. The full analytic cohort (N = 1,167) was used with the four-level clinical outcome (conservative management, negative CT, clinical TBI diagnosis, CT-confirmed TBI) and the same 10 variables used in the primary analysis: loss of consciousness, emesis, headache, severe mechanism of injury, hematoma, PECARN high-risk status, PECARN low-risk status, English-speaking status, White race, and continuous age. Models requiring scaling used center-and-scale preprocessing within each cross-validation fold. XGBoost was excluded from the final comparison due to degenerate probability estimates across all hyperparameter combinations.

The six models compared were: multinomial logistic regression (L2 regularization, decay tuning), elastic net regularized multinomial regression, random forest (500 trees), gradient-boosted trees (excluded as noted), radial-basis support vector machine, and k-nearest neighbors. The primary performance metric was multiclass log loss; secondary metrics included multiclass area under the receiver operating characteristic curve (AUC), accuracy, Cohen's kappa, and mean balanced accuracy.

Mean performance across 50 resamples is shown in the table below. The two linear models — multinomial logistic regression and elastic net — performed equivalently and outperformed all nonlinear alternatives. The log loss difference between elastic net and multinomial logistic regression was 0.002, and the optimal elastic net used minimal regularization (α = 0.1, λ = 0.016), indicating the multinomial model does not meaningfully overfit. Random forest, which can detect arbitrary interactions among variables, performed worse than both linear models, providing no evidence that nonlinear relationships or interaction effects between clinical and demographic variables improve estimation of clinical pathway.

| **Model** | **Log Loss** | **Accuracy** | **AUC** | **Kappa** |
| --- | --- | --- | --- | --- |
| Elastic net | 0.858 (0.027) | 0.673 (0.015) | 0.775 (0.017) | 0.323 (0.035) |
| Multinomial logistic | 0.860 (0.026) | 0.675 (0.017) | 0.773 (0.018) | 0.349 (0.039) |
| Random forest | 0.875 (0.025) | 0.675 (0.017) | 0.767 (0.020) | 0.333 (0.037) |
| SVM (radial) | 0.951 (0.024) | 0.652 (0.019) | 0.694 (0.023) | 0.273 (0.038) |
| KNN | 1.142 (0.267) | 0.666 (0.020) | 0.761 (0.020) | 0.315 (0.040) |

*Values are mean (SD) across 50 resamples.*

Per-class one-vs-rest AUC was strongest for CT-confirmed TBI (0.849) and conservative management (0.822), and weakest for negative CT (0.651), the most discretionary outcome. Variable importance was dominated by clinical symptom variables across all model types; demographic variables contributed minimally (English-speaking: 1.3% in multinomial, 0.7% in random forest; White race: 0.0% in multinomial, 0.9% in random forest), with no evidence of nonlinear demographic-clinical interactions in tree-based models.

Analyses were performed in R 4.4.1 via Altair Analytics Workbench (PROC R) using the caret package (version 7.0-1). Additional packages: nnet 7.3-20, glmnet 4.1-10, ranger 0.18.0, kernlab 0.9-33, e1071 1.17-17.

**Classification Tree: Conditional Decision Structure**

Supplemental Figure 3 illustrates the conditional decision structure among intermediate-risk patients as an exploratory complement to the regression. OSS was the most influential feature (importance

0.62), with the initial split at OSS ≤ 1 versus OSS ≥ 2 dividing patients into a Clinical Judgment Zone (n = 260) and an Imaging Zone (n = 170) with markedly different CT rates (23% vs 71%; P < .001).

Within the Clinical Judgment Zone, headache was the first subsequent split, followed by age and language preference. Among children aged ≤11 years with headache, the tree separated English-speaking (Leaf B; n = 131) and non-English-speaking patients (Leaf C; n = 29). Non-English-speaking patients in this branch had a higher proportion of clinical TBI diagnosis (38% vs 23%) and lower CT rate (10% vs 22%) than English-speaking patients, though the difference was not statistically significant (P = .16). The direction was consistent with the adjusted regression finding.

Within the Imaging Zone, CT was the majority pathway across all terminal nodes. Among patients without headache, the tree split at age 3 years; children aged ≤3 years (Leaf F; n = 38) had a CT-positive yield of 27%, significantly lower than older children in the same branch (78%; P < .001) and the only terminal node significantly below the high-risk reference yield of 62% (Dunnett P < .05). Among patients with headache in the Imaging Zone, language preference produced an additional split; CT was performed in 68% of non-English-speaking patients (Leaf J; n = 22) compared with 77–85% of English-speaking patients in analogous branches.
