# Supplementary material for: English versus Non-English Language Preference and Computed Tomography Use in Pediatric Minor Head Trauma
Source: West J Emerg Med. 2026 Oct 26;27(6):1660–70. doi: 10.5811/westjem.64410 (PMC13762077; doi:10.5811/westjem.64410)
Supplement: Supplementary file 2 [file wjem-27-1660-s002.docx]

**Supplemental Table 1.** ICD-10 diagnosis codes used to identify eligible patients in a retrospective cohort study of pediatric minor head trauma at a single-institution pediatric emergency department, 2019–2022. Codes required primary diagnosis designation and align with the Centers for Medicare and Medicaid Services Merit-based Incentive Payment System Clinical Quality Measure for emergency department CT utilization in minor blunt head trauma (Quality ID #416). Codes are organized by anatomical region.

*CT, computed tomography; ICD-10, International Classification of Diseases, Tenth Revision.*

| Category | ICD-10 Codes |
| --- | --- |
| Scalp & Face Wounds (S00) | S00.03XA, S00.33XA, S00.431A, S00.432A, S00.439A, S00.531A, S00.532A, S00.83XA, S00.93XA |
| Skull & Cranial Bones (S02) | S02.0XXA, S02.101A, S02.102A, S02.109A, S02.110A–S02.11HA (all variants), S02.19XA, S02.2XXA, S02.30XA–S02.32XA, S02.400A–S02.413A, S02.42XA, S02.600A–S02.672A, S02.69XA, S02.80XA–S02.92XA |
| Intracranial Injury (S06) | S06.0X0A–S06.0X9A, S06.1X0A–S06.1X9A, S06.2X0A–S06.2X9A, S06.300A–S06.369A, S06.4X0A–S06.4X9A, S06.5X0A–S06.5X9A, S06.6X0A–S06.6X9A, S06.810A–S06.894A, S06.9X0A–S06.9X9A |
| Other Head/Neck Trauma (S09, S10) | S09.10XA, S09.11XA, S09.19XA, S09.8XXA, S09.90XA, S09.92XA, S09.93XA, S10.0XXA, S10.83XA, S10.93XA |
