# Supplementary material for: English versus Non-English Language Preference and Computed Tomography Use in Pediatric Minor Head Trauma
Source: West J Emerg Med. 2026 Oct 26;27(6):1660–70. doi: 10.5811/westjem.64410 (PMC13762077; doi:10.5811/westjem.64410)
Supplement: Supplementary file 3 [file wjem-27-1660-s003.docx]

**Supplemental Table 2.** Individual clinical sign associations with clinical pathway from multinomial logistic regression among intermediate-risk pediatric patients with minor head trauma at a single-institution pediatric emergency department, 2019–2022 (N = 430). Reference category: no CT and no TBI diagnosis (n = 162).

|  | **TBI without CT (n=88)** |  | **CT performed (n=180)** |  |
| --- | --- | --- | --- | --- |
| **Variable** | **OR (95% CI)** | **P** | **OR (95% CI)** | **P** |
| Headache | 4.48 (1.39–14.47) | .01 | 2.50 (1.22–5.14) | .01 |
| Age (per 5-year increase) | 1.70 (1.14–2.54) | .01 | 1.20 (0.85–1.71) | .31 |
| Non-English-speaking | 1.27 (0.68–2.38) | .45 | 0.47 (0.24–0.92) | .03 |
| Emesis | 2.21 (0.88–5.54) | .09 | 16.18 (7.83–33.43) | < .001 |
| Loss of consciousness | 0.59 (0.15–2.35) | .46 | 9.86 (4.40–22.12) | < .001 |
| Hematoma | 0.87 (0.26–2.95) | .82 | 3.71 (1.56–8.82) | .003 |
| Severe mechanism of injury | 0.94 (0.33–2.63) | .90 | 3.24 (1.48–7.05) | .003 |

*aOR, adjusted odds ratio; CI, confidence interval; CT, computed tomography; TBI, traumatic brain injury.*
